# Supplementary material for: An open-source framework for large-scale, flexible evaluation of biomedical text mining systems
Source: J Biomed Discov Collab. 2008 Jan 29;3:1. doi: 10.1186/1747-5333-3-1 (PMC2276192; doi:10.1186/1747-5333-3-1)
Supplement: Additional file 1 — Evaluation platform source code. The additional file contains the software evaluation framework discussed in this paper. The most current release can be downloaded from SourceForge.net [1]. See the accompanying README file for instructions on installation and use. Also included in the distribution is a collection of UIMA wrappers for some commonly used BioNLP tools and annotated corpora. [file 1747-5333-3-1-S1.zip › bionlp_uima_repository-1.0.1/docs/api/src-html/edu/uchsc/ccp/uima/annotation/syntactic/CCPSentenceAnnotation.html]

```
001    
002    
003    /* First created by JCasGen Tue Aug 28 16:44:10 MDT 2007 */
004    package edu.uchsc.ccp.uima.annotation.syntactic;
005    
006    import org.apache.uima.jcas.JCas;
007    import org.apache.uima.jcas.JCasRegistry;
008    import org.apache.uima.jcas.cas.TOP_Type;
009    
010    import edu.uchsc.ccp.uima.annotation.CCPSyntacticAnnotation;
011    
012    
013    /** This annotation type is used to annotate sentences within the document text.
014     * Updated by JCasGen Tue Aug 28 16:44:10 MDT 2007
015     * XML source: /home/williamb/eclipse/RELEASED_SOURCE_CODE/descriptors/typeSystem/CCPTypeSystem.xml
016     * @generated */
017    public class CCPSentenceAnnotation extends CCPSyntacticAnnotation {
018      /** @generated
019       * @ordered 
020       */
021      public final static int typeIndexID = JCasRegistry.register(CCPSentenceAnnotation.class);
022      /** @generated
023       * @ordered 
024       */
025      public final static int type = typeIndexID;
026      /** @generated  */
027      public              int getTypeIndexID() {return typeIndexID;}
028     
029      /** Never called.  Disable default constructor
030       * @generated */
031      protected CCPSentenceAnnotation() {}
032        
033      /** Internal - constructor used by generator 
034       * @generated */
035      public CCPSentenceAnnotation(int addr, TOP_Type type) {
036        super(addr, type);
037        readObject();
038      }
039      
040      /** @generated */
041      public CCPSentenceAnnotation(JCas jcas) {
042        super(jcas);
043        readObject();   
044      } 
045    
046      /** @generated */  
047      public CCPSentenceAnnotation(JCas jcas, int begin, int end) {
048        super(jcas);
049        setBegin(begin);
050        setEnd(end);
051        readObject();
052      }   
053    
054      /** <!-- begin-user-doc -->
055        * Write your own initialization here
056        * <!-- end-user-doc -->
057      @generated modifiable */
058      private void readObject() {}
059         
060    }
061    
062
```
